# Implementing a One Health Approach to Rabies Surveillance: Lessons From Integrated Bite Case Management

**DOI:** 10.3389/fitd.2022.829132

**Published:** 2022-06-20

**Authors:** Catherine Swedberg, Stella Mazeri, Richard J. Mellanby, Katie Hampson, Nai Rui Chng

**Affiliations:** 1 Institute of Biodiversity, Animal Health and Comparative Medicine, University of Glasgow, Glasgow, United Kingdom; 2 Royal (Dick) School of Veterinary Studies and the Roslin Institute, University of Edinburgh, Easter Bush Campus, Roslin, United Kingdom; 3 Institute of Health and Wellbeing, College of Medical, Veterinary and Life Sciences, University of Glasgow, Glasgow, United Kingdom

**Keywords:** dog-mediated rabies, rabies elimination, rapid diagnostic tests, mobile health, post-exposure prophylaxis, zoonosis, implementation research

## Abstract

As part of the ‘Zero by 30’ strategy to end human deaths from dog-mediated rabies by 2030, international organizations recommend a One Health framework that includes Integrated Bite Case Management (IBCM). However, little is understood about the implementation of IBCM in practice. This study aims to understand how IBCM is conceptualized, exploring how IBCM has been operationalized in different contexts, as well as barriers and facilitators to implementation. Semi-structured interviews were conducted with seventeen practitioners and researchers with international, national, and local expertise across Africa, Asia, and the Americas. Thematic analysis was undertaken using both inductive and deductive approaches. Four main themes were identified: 1) stakeholders’ and practitioners’ conceptualization of IBCM and its role in rabies elimination; 2) variation in how IBCM operates across different contexts; 3) barriers and facilitators of IBCM implementation in relation to risk assessment, PEP provisioning, animal investigation, One Health collaboration, and data reporting; and 4) the impact of the COVID-19 pandemic on IBCM programs. This study highlights the diversity within experts’ conceptualization of IBCM, and its operationalization. The range of perspectives revealed that there are different ways of organizing IBCM within health systems and it is not a one-size-fits-all approach. The issue of sustainability remains the greatest challenge to implementation. Contextual features of each location influenced the delivery and the potential impact of IBCM. Programs spanned from highly endemic settings with limited access to PEP charged to the patient, to low endemicity settings with a large patient load associated with free PEP policies and sensitization. In practice, IBCM was tailored to meet the demands of the local context and level of rabies control. Thus, experts’ experiences did not necessarily translate across contexts, affecting perceptions about the function, motivation for, and implementation of IBCM. To design and implement future and current programs, guidance should be provided for health workers receiving patients on assessing the history and signs of rabies in the biting animal. The study findings provide insights in relation to implementation of IBCM and how it can support programs aiming to reach the Zero by 30 goal.

## Introduction

Effective rabies vaccines for humans and animals have been available for over a century, providing means to eliminate this fatal and incurable zoonotic disease (1). Through mass dog vaccinations starting in the 1920s, rabies has been eliminated from domestic dog populations in high-income countries across western Europe, North America, and parts of Asia, such as Japan and Taiwan (2, 3). Additionally, low- and middle-income countries (LMICs) in Latin America have successfully reduced dog-mediated rabies cases by over 98% through large-scale coordinated dog vaccination programs (4, 5). Despite this progress, an estimated 59,000 people still die from rabies every year, with the vast majority in resource-poor countries in Africa and Asia (2, 6).

There are several challenges for LMICs in reducing the burden of rabies within their populations. First, to control and eliminate rabies a high vaccination coverage, typically around 70% of the susceptible dog population, must be sustained for 3-7 years *via* recurrent annual campaigns (2). Yet many LMICs have not initiated routine dog vaccination (7). Furthermore, countries face the threat of reintroductions if endemic rabies circulates at their borders (8). Second, post-exposure prophylaxis (PEP)—which is needed immediately after a bite from a rabid dog to prevent the fatal onset of rabies—is often expensive for both bite victims and governments. As a result, PEP availability is frequently limited, especially in rural areas (9, 10). PEP costs can even drain the finite budget available for rabies, without impacting the incidence of rabies in dog populations that are the source of exposures (11, 12). Third, surveillance in LMICs is typically weak and does not capture accurate data on either human or animal rabies cases (13). This significant under-reporting leads to lack of awareness and understanding of the burden of rabies, which further results in limited community/stakeholder engagement and inadequate funding. Thus, the absence of robust surveillance gives rise to a cycle of underestimating the disease burden and consequently neglecting control measures, such as dog vaccination and PEP provisioning (12, 14).

To overcome these challenges, the Tripartite [World Health Organization (WHO), World Organisation for Animal Health (OIE), Food and Agriculture Organization (FAO)] along with the Global Alliance for Rabies Control (GARC) developed the ‘Zero by 30’ global strategic plan to end human deaths from dog-mediated rabies by 2030. Within this strategy, international organizations jointly recommend a One Health framework, recognizing the interconnections between the health of humans, animals, and their shared environment (11). To control and eliminate rabies, the strategy advocates Integrated Bite Case Management (IBCM) (15). WHO describes IBCM as an advanced surveillance method involving “investigations of suspected rabid animals and sharing information with both animal and human health investigators for appropriate risk assessments” (2). Through multisectoral collaboration and communication, this One Health approach aims to enhance surveillance by increasing detection of animal cases and human exposures, as well as to improve PEP allocation and compliance (4, 13, 16).

While the objectives, aims, and benefits of IBCM are becoming better known by international organizations and experts in the field, this approach is relatively new and has only been implemented within the last decade. Official guidelines for IBCM and risk assessments in relation to biting animals were first mentioned in WHO guidance in 2018 (2). Peer-reviewed empirical evidence about the impact or implementation of IBCM remains limited. A PubMed search using keywords “novel surveillance” OR “integrated bite case management” AND “rabies” identified only eleven studies from four countries: Chad (10, 17), Haiti (4, 14, 16, 18–20), the Philippines (12, 21), and Tanzania (13). Although several IBCM programs have been implemented within the last decade, the approaches undertaken and the lessons learned from these programs have not yet been synthesized. This study aims to understand how IBCM is conceptualized and practiced by stakeholders involved in rabies prevention and control programs and barriers and facilitators to its implementation.

## Materials and Methods

This qualitative study was conducted among experts with experience designing, implementing, and/or managing IBCM programs in different epidemiological and geographical contexts (Table 1). Purposive sampling of known professional networks was used to identify and recruit experts. All participants were contacted by email and provided with a participant information sheet. Participants provided consent prior to interviews, which were all conducted in English.

Seventeen participants were interviewed: five international-level, six national-level, and six local-level experts. The majority of participants had a degree in veterinary medicine (twelve); a doctoral research degree (nine); or both (five); and one had a MSc in medical statistics. All participants had some educational/experiential background in epidemiology. Their work experience ranged from academic, government, non-profit, and international organization, with most having experience with more than one. Fourteen IBCM programs were included in the study representing thirteen countries in the Americas, Africa, and Asia (Table 2). All IBCM programs were/are being implemented in countries with endemic dog rabies, with the exception of Rio Grande Do Sul, a state in Brazil which has not reported a dog or human case since the 1980s.

Semi-structured one-to-one interviews (40-65 minutes) were conducted between January 2020 and August 2021. One interview was conducted face-to-face before COVID-19 restrictions and the other sixteen were conducted over the videoconferencing platform, Zoom (49). Interview topic guides were generated for each level of expertise: international, national, and local. The questions were designed to be open-ended and encourage experts to share their experiences and elaborate on the issues they felt to be important. Interviews were audio-recorded, transcribed verbatim, pseudonymized, then uploaded into NVivo 12 Pro software (50).

Data analysis was conducted by the first author and supervised by the last author, an experienced qualitative researcher. The data was analyzed using a six-step thematic analysis (51). All transcripts were read for familiarization to develop initial codes. An inductive approach was used to develop descriptive codes identified from similar patterns, topics, and elements of the intervention, which were then collated into themes, categories, and subcategories. Transcripts were also coded deductively using assumptions underlying a logic model of IBCM, depicting the relationship between program activities and the intended impact of IBCM. Themes were developed and reviewed iteratively and checked for consistency and appropriateness, amending where necessary. Themes included: inputs, activities, outputs, outcomes, and aims. Transcripts were compared for differences and similarities in how IBCM was operationalized, barriers and facilitators encountered during implementation, and the desired outcomes and aims of each program. Interviewees were sent a copy of the manuscript to validate accurate representation of their IBCM program and their feedback was incorporated.

## Results

### Conceptualization of IBCM

#### Description of IBCM

Several experts first heard the term ‘Integrated Bite Case Management’ in relation to rabies control in Bali, Indonesia in 2011 (52). However, most learned of IBCM from a program in Haiti (14) or through their own experience. All programs used the term IBCM, except for three where their intervention was referred to as either ‘a One Health approach to bite management’ (Chad) (10); ‘clinic-based surveillance’ (Madagascar) (27); or a ‘One Health approach’ to guide PEP recommendations (Brazil) (31).

Experts described IBCM in a way that combined its key components (activities) with the role it plays (outputs/outcomes):

“IBCM at its simplest is the ability to provide a proper risk assessment, usually in the context of the exposing animal, in a way in which the outcomes of the risk assessment can impact the human treatment decision.” (Expert #1, International-level)

**Figure 1:**
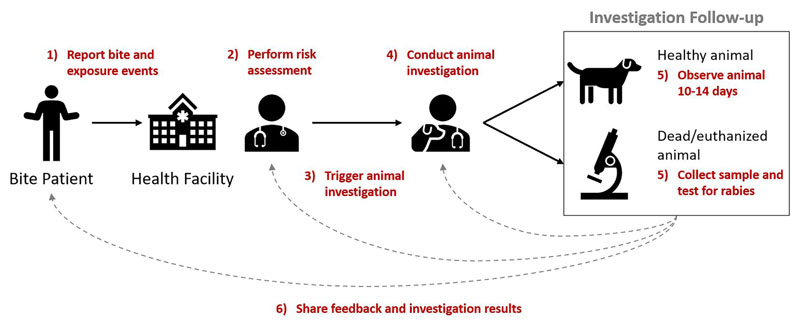
Key components of IBCM. Annotated in red are the six components (activities) that comprise the IBCM approach with arrows and numbering indicating the sequential order of these components.

These reported components (activities) were mostly consistent and aligned with the WHO definition of an IBCM program. They included: 1) reporting a bite or exposure event, 2) performing a risk assessment, 3) triggering an investigation for any bite deemed high-risk, 4) conducting an animal investigation, 5) observing animal for 10-14 days (to confirm a healthy animal) or collecting samples and diagnostic testing (from dead/euthanized animals), and 6) sharing feedback and investigation results across sectors (Figure 1).

Although a consensus emerged about the required components of IBCM, there was still some uncertainty about the definition. Specifically, experts had varied opinions about whether IBCM must always be initiated by a bite event, or determine treatment decisions or be used to specifically enhance surveillance. Therefore, while most interviewees perceived their work as being IBCM or similar to IBCM, some did not consider their program to formally be IBCM (for example, in Southern Brazil where the objective was not to strengthen surveillance, but to better manage PEP).

Participant’s concept of IBCM evolved over time and with experience. Most international-level experts viewed this approach as “passive public health surveillance” initiated by any suspect rabid animal – not only from bites:

“I used to think it was integrated BITE case management. Whereas, my attitude now is it’s the full investigation of a suspect animal, whether it’s for a bite or just a dog in the community behaving strangely.” (Expert #3, International-level)

#### Purpose of IBCM

**Figure 2:**
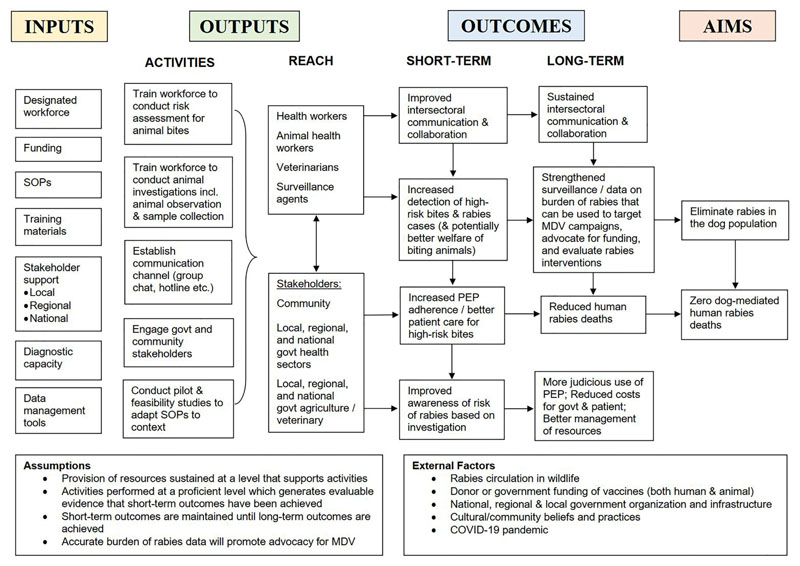
Logic model of IBCM. Representation of the relationship between resources (inputs), activities, outputs, short- and long-term outcomes, and aims of an IBCM program.

Participants consistently identified several key roles of IBCM (outlined in the outcomes section of our conceptualized logic model, Figure 2). These roles were emphasized differently for each program and not all roles were relevant for every program. These roles were to: a) enhance surveillance through improved case detection, thereby enabling evaluation of control and prevention measures; b) directly and formally connect the health sector to the veterinary sector; c) inform PEP administration, aiming to improve patient care and increase adherence; d) better manage limited resources through judicious use of PEP; and e) advocate for community and stakeholder support and funding for rabies programs, and guide their implementation.

Experts agreed that countries with endemic dog rabies should all have a surveillance program, but opinions differed on when IBCM should be incorporated. A few experts viewed IBCM as an advanced surveillance strategy specifically meant for countries with a well-established control program who were close to elimination. Others argued that IBCM is fundamental and required at all stages (e.g., endemic, emerging, elimination and post-elimination) within routine surveillance:

“IBCM is needed as a country scales up its rabies elimination efforts and as an early intervention to try to bring down human deaths. It’s needed in countries where 1) you have a lot of human deaths and you need to do a better job getting PEP to the people at risk and 2) as you really start to take elimination seriously, it’s needed as a foundational system for evaluating the efforts that are going into vaccinating dogs. Then it’s important in the end-game, post-elimination phase to continue to evaluate the risk to people bitten.” (Expert #3, International-level)

### Operationalization of IBCM

There was considerable variation in how IBCM was operationalized across settings, which were diverse in terms of their economic and epidemiological contexts (Table 1).

The most important prerequisites considered necessary for IBCM were the identification of a designated person/team responsible for investigating animals; and health facilities (hospitals, clinics, etc.) where bites are reported and PEP is administered. Several experts further mentioned the importance of stakeholder and community engagement prior to implementation.

The key input that differed between programs was who was identified and trained to carry out activities (Table 2). Three categories of workforce were identified: fully hired, a combination of hired and local government, and fully local government. Other inputs that varied were the use of rapid diagnostic tests (RDTs) and mobile applications for reporting/data management.

Program activities were similar amongst each workforce category. IBCM programs with a fully hired workforce—meaning their primary job responsibility was rabies/IBCM and their salary was paid by external funding—relied on the same team or person to conduct most or all of the IBCM components:

“We have trained surveillance agents … They all have the app on a tablet and go to the sentinel hospitals in their area weekly to check for bites … also through word of mouth. Then they go out and investigate the dog bites and report them.” (Expert #10, National-level)

In contrast, programs with a fully local government workforce trained existing capacity to complete IBCM activities. This involved health workers (nurses, doctors, etc.) conducting risk assessments and alerting their animal health counterparts (animal health workers, veterinarians, etc.) to investigate biting animals. Programs with a combined workforce trained a hired team and used local government staff—sometimes volunteer medical/veterinary students—to conduct risk assessments or animal investigations.

Almost all IBCM programs used paper-based or electronic registers from health facilities to collect bite data. These were typically from district and regional-level hospitals supplying PEP, but in some countries from rural community-level clinics with PEP access (e.g., Philippines). In addition to registers from health facilities, some programs used hotlines and/or trained local community health workers to report bite events. This was done particularly to enhance surveillance in rural areas with low PEP-seeking behaviors or limited access to PEP.

The mechanism to trigger animal investigations varied from calling/messaging the investigators, using hotline staff to notify them, and/or group chats or submitting data into mobile apps that send notifications to investigators.

### Barriers and Facilitators to IBCM Implementation

Reported barriers and facilitators could be placed under five main categories: risk assessment; PEP provisioning; animal investigation; the use of IBCM to facilitate One Health collaboration; and data reporting and mobile technology (Table 3).

#### Risk Assessment

IBCM programs with a hired workforce designated to perform risk assessments generally experienced fewer challenges than those with a local government workforce. Health workers were often stretched by busy workloads and other responsibilities/priorities. Some commonly reported barriers were: high volume of patients; added workload without compensation; high staff turnover; feeling that IBCM is not their responsibility or lacking interest; frustrations from work duplication (already have a reporting system); no accountability or lack of supervisory support; and reluctance to change/adopt a new way of working. Hiring staff addressed many of these barriers since rabies was their primary responsibility. Though this usually required additional funding from research grants or donors, challenging sustainability.

Some programs aimed to use risk assessments for more judicious use of PEP, typically in locations with frequent shortages or high expenditure on rabies biologics. These programs often experienced the challenge of ensuring health workers could perform risk assessments to a sufficiently reliable and effective level for PEP to be withheld or discontinued. While some health workers were proficient using WHO protocols to assess wound severity (Category I, II, and III), others required multiple training sessions. The majority were not familiar with the clinical signs of rabies in animals or the importance of assessing risk *via* the status of the biting animal:

“The main issue is that it’s been difficult for the nurses to perform high-risk assessments … a lot of the cases they post in the peer support chat are not high-risk based on our definition. And when you look at the protocols of the Department of Health, they actually have a lot more criteria on what is considered high-risk, which concentrates on the nature of the wound rather than the animal status.” (Expert #15, Local-level)

Other significant challenges regarding judicious use of PEP included there being no legal basis for determining PEP decisions from risk assessments; and health workers’ hesitancy to withhold or discontinue PEP, even where there was no apparent risk. Hard copy protocols, routine training and communication, and use of apps that automatically assign the animal case definition, supported health workers’ ability to more accurately determine low-risk vs high-risk bites and make treatment decisions.

#### PEP Provisioning

Most experts expressed that the level of PEP provisioning influenced health-seeking behavior, adherence, and the number of bite patients that present to health facilities. Free PEP policies reportedly increased accessibility and adherence to vaccine regimens. However, the provision of free PEP could also lead to much higher patient throughput and sometimes an excessive workload for those performing risk assessments. Furthermore, demand for PEP frequently remained high even when the risk of rabies was very low or even zero.

Typically, health-seeking and PEP adherence is much lower where patients pay for PEP or where travel costs are high due to limited PEP accessibility and availability. These settings often have a lower throughput of patients, with a larger proportion of those presenting as very high risk, as patients make their own assessment of risk. A few experts said that despite limited access to PEP being an issue, IBCM was often easier to implement in these settings:

“Of the people presenting for post-exposure prophylaxis, we’re probably looking at about half of them being bitten by dogs that we would consider to be high-risk, and likely to be rabid. We don’t see a lot of patients, but the patients that we see have a high chance of being really at risk for rabies.” (Expert #5, International-level)

Experts reported that the most-at-risk regions for rabies were rural areas where people had poor access to health services and may seek out traditional healers after being bitten. This also may affect the performance of health facilities supplying PEP as sentinels for high-risk bites. Some experts stated that improving PEP access could use up substantive rabies funding with diminishing returns. Alternatively, they felt that investment in mass dog vaccination, could decrease the incidence of rabies in source populations.

#### Animal Investigation

Barriers to conducting animal investigations using a government workforce were similar to those reported for risk assessments. Oftentimes, when an investigation was triggered, they were not conducted, were conducted too late, or there was no follow-up after the 10-14 day observation period. Factors included: required time/travel/resources (fuel, transport etc.); lack of personnel; high staff turnover; prioritizing diseases considered more economically important in livestock and poultry (e.g., foot- and-mouth disease, avian influenza, lumpy skin disease); lacking formal training as a veterinarian; and/or not feeling comfortable handling animals. Commonly, the person designated to investigate the animal had a background and responsibilities unrelated to animal rabies control and did not feel rabies was their job:

“The problem is that these inspectors can be veterinarians, biologists, environmental engineers or what they call ‘technicians’ without formal training … When they’re biologists, they spend most of their time doing vector surveillance … If they’re environmental scientists and engineers, they’re more interested in water or restaurant inspection … When there is a veterinarian, usually there is more focus on rabies.” (Expert #9, National-level)

Hiring program staff resolved many of these issues, but was not always an option due to funding constraints. Hired investigators also reportedly felt more confident handling animals and collecting samples, although hands-on training improved these skills for government investigators. Both hired and government staff experienced similar challenges during investigations, including not being able to find or identify the biting animal or collect a sample because the animal was already killed, buried, or decomposing on investigation.

A few countries had only one diagnostic laboratory with limited capacity in terms of equipment, staff, and quality control. Samples sometimes had to be shipped long distances, without cold chain or costs covered, often limiting sample submission to nearby areas. IBCM programs implemented in locations with established diagnostic capacity had a significant advantage. Furthermore, programs that trained field staff to use hook and straw sampling techniques simplified procedures and facilitated the collection, storage, and shipping of animal samples.

Some experts stated that rapid diagnostic tests (RDTs) are not reliable and pose complications to protocols for treatment decisions in the case of false negative results. Moreover, RDTs are not yet recommended by WHO and OIE, thus there is no guidance available for practitioners. Other experts found RDTs to be a facilitator for implementing IBCM, encouraging investigators to collect samples by providing immediate results to report to the health sector and communities:

“The vets applied rapid test kits, which are not validated yet, but it was very good to give the vets something at hand to empower them. The veterinary system is usually less financed than the human health services. If you want to apply ‘One Health’ you should empower them to bring them closer to the human health services. Otherwise, it’s a mismatch between roles … This test balanced it out a bit by giving the vets something to motivate the human health services to communicate with them.” (Expert #7, National-level)

Protocols usually stipulated that all samples tested with RDTs should be confirmed with laboratory diagnostics and that PEP decisions should not depend on RDT results. Experts stated that RDT results generally matched laboratory results.

Experts noted that motivation to investigate commonly increased only after a human death or several animal cases in their area. Moreover, IBCM typically increased detection resulting in a swift rise in cases, which could be a disincentive, especially when approaching elimination. Sometimes animal investigators would be blamed for rising cases, which discouraged their future reporting. Experts articulated the importance of preparing leadership to expect increased case numbers on introduction of IBCM and explain that this provides better guidance for control.

#### The Use of IBCM to Facilitate One Health Collaborations

Barriers to collaboration between sectors were found at the national, regional, and local level. Government ministries were rarely structured to facilitate intersectoral collaboration and faced challenges getting sectors to work together. Typically, sectors had unbalanced power, with the human health sector having more resources in terms of funding and influence. The priority placed on national rabies programs varied between countries, and was often neglected by both sectors. Using pre-existing One Health programs as a resource for IBCM activities was reported as a significant advantage. Also, programs that involved all relevant sectors in joint discussions, decisions, and training experienced more success:

“You need to have the buy-in of both the health and veterinary sectors, from the national- to the local-level. Because if they don’t think it’s important then we cannot force them to implement IBCM. They have to have a better understanding of why it is important so that it translates to actual work. If the National Program doesn’t believe in it, then it is pointless to push it further. But if they recommend IBCM as a policy, then it should go down the line … from national, regional, to local government.” (Expert #2, International-level)

Experts talked about the difficulty of creating local ownership and changing routine behaviors for information sharing at the individual level up to the ministerial level. There were barriers to getting the health sector to report high-risk bites to their animal health counterparts and to consider investigation results when making treatment decisions. Establishing and maintaining a line of communication between sectors was challenging in many settings. In a few instances, local government staff had limited data management skills which made it hard to link human bite cases to animal investigations.

To overcome these challenges, experts emphasized the importance of providing regular feedback and establishing a rapport between human and animal health workers, IBCM staff, and other stakeholders. Additionally, experts reported the need to consider local context and adapt protocols accordingly. Innovations, such as using hotlines as the link between sectors and the community, or developing different protocols for urban and rural settings, encouraged participation. Lastly, a few countries facilitated One Health collaboration by employing veterinary staff within the public health system (e.g., Brazil) - a strategy used in many high-income countries (e.g., United States).

#### Data Reporting and Mobile Technology

Experts reported a common barrier was lack of data submission from all involved sectors. Many times, the protocols for the risk assessment and all steps of the investigation (e.g., quarantine, follow-up, sample collection) were completed, but results were not reported, nor was feedback provided to other sectors.

The use of mobile applications for data reporting and management created both challenges and opportunities. App development initially took a great deal of time and many iterations. Yet once finalized, apps enabled IBCM protocols to be standardized by providing a template for questions and procedures for the risk assessment and animal investigation. Experts said the app allowed real-time data to be accessed remotely by program managers. This facilitated rapid identification and response to high-risk cases, as well as the timely provisioning of information to stakeholders and communities:

“The apps allow the technical expertise to sit anywhere in the world and real-time monitor cases, [human] vaccinations, dog vaccination programs … In the past it would take 2-3 years to get all of this paper data, enter it, and start to learn anything from your system. This ability to real-time evaluate and monitor what’s going on and make adjustments is invaluable.” (Expert #1, International-level)

While programs using mobile apps experienced issues with network coverage and internet access, these barriers were overcome with a feature allowing data to be saved. Not all program staff had access to smartphones where the app could be downloaded, which required some programs to purchase tablets or work phones. In addition, experts mentioned there was reluctance from some staff to use the app. Older staff in particular experienced difficulties using this technology and often required additional proficiency training. Changes in technology meant apps frequently needed to be re-downloaded/updated or risked becoming obsolete.

Most experts felt apps were a solution to many issues, while others saw them as an added complexity to training and implementation. In many settings, mobile apps helped overcome language barriers and could be tailored to local contexts (language, geo-hierarchy etc.). Yet one expert said using a mobile app would be difficult in their setting due to high illiteracy in official and local languages.

### Impact of COVID-19

IBCM programs encountered COVID-19-related obstacles on several levels. Some program start dates were postponed, and most training for ongoing programs were postponed or canceled in 2020 and 2021. Moreover, allocated time and resources (personnel, diagnostics, vaccine storage, surveillance efforts, supply chains, funding) were re-focused on COVID-19:

“I think the main impact that COVID-19 is going to have on rabies in [country] is that it’s going to hide the problem. Because the surveillance system stops - it’s now focused on COVID … most of the resources for the lab go to COVID. And let’s say on a regular basis rabies is really neglected - now it’s going to be even more neglected.” (Expert #11, National-level)

Increased pressure on the health sector meant many health workers were extremely busy and did not report high-risk bites as frequently. Local travel restrictions limited in-person animal investigations or prevented them entirely. Experts in some countries observed drops in animals tested due to decreased surveillance efforts. Several IBCM programs experienced declines in bite patient presentations, typically for 2-5 months at the start of 2020 lockdowns. Experts similarly described the cancellation or disruption of dog vaccination campaigns in 2020 and/or 2021 resulting in lower vaccination coverages. Some areas are already seeing rising human and dog cases, further necessitating the need for IBCM.

Lastly, the pandemic affected peoples’ livelihoods and caused income losses, making healthcare less affordable. Experts speculated that people are not feeding community dogs as frequently, leading them to roam farther for food and creating a more favorable environment for rabies transmission. People were reported to have also been abandoning pets due to costs and fear of COVID-19 transmission, potentially increasing stray dog numbers. In order to accurately measure the impact of the pandemic, enhanced surveillance such as IBCM is needed.

## Discussion

The main finding from this study was the variation between IBCM programs reported across epidemiological and geographical settings. Specifically, the interviews highlighted the diversity within experts’ conceptualization of the definition and roles of IBCM. This range of perspectives demonstrates that there are different ways of organizing IBCM within health systems and that it is not a one-size-fits-all approach. While there was consensus among experts and the wider literature about the required components (inputs and activities) of IBCM (Figure 1) which aligned with WHO guidance (2), these components were operationalized differently between programs. Moreover, experts’ perspective of the purpose of IBCM often differed and by implication, so did the desired outcomes of each program. In practice, IBCM was tailored to meet the demands of the local context and level of rabies control in place. Experts were well versed about how IBCM operated in their settings and what outcomes they wished to achieve by implementing this One Health approach. But experiences did not necessarily translate across contexts, affecting perceptions about the function, motivation for, and implementation of IBCM. Nonetheless, despite differences in operationalization and desired outcomes, programs shared many similar experiences with the challenges they faced and progress in overcoming them.

Contextual features of each location—which can be described broadly as epidemiological or non-epidemiological (53)—contributed to differences in desired outcomes and barriers and facilitators to implementation. The epidemiological context (e.g., human deaths, incidence in dog population) partially influenced how much rabies was prioritized at the national and local level. However, most variation in terms of the success of IBCM and the impact achieved was due to the non-epidemiological context. This includes features such as: social and economic (e.g., health-seeking behaviors, GDP, HDI); cultural (beliefs, attitudes, and practices among policymakers, practitioners, communities); geographical (e.g., urban vs. rural); service and organization (e.g., motivation, willingness to change); policy (PEP provisioning); financial (e.g., funding); political (e.g., level of decentralization, distribution of power among sectors/stakeholders); and historical (e.g., presence of rabies). The issue of sustainability was at the core of many barriers to implementation, which the literature acknowledges as a major hindrance for other evidence-based interventions (54). Using an existing government workforce for program activities was typically seen as more sustainable. Yet, experts found it difficult to incentivize or motivate human and animal health workers to change their way of working and complete extra work without supplemental pay. The overall success of programs appeared to be influenced by practitioners’ and stakeholders’ viewpoint of the added value of IBCM relative to the extra time and effort required from them and ultimately the degree to which funding was allocated.

Most programs operated in settings with endemic dog rabies, weak or nonexistent surveillance, and scarce funding. Thus, the primary desired outcome was to establish a cost-effective surveillance system to rapidly identify high-risk human exposures and potential rabid animals. One exception was the IBCM-like approach used in three southern states in Brazil, where dog rabies has been eliminated and strong surveillance already exists. Instead of surveillance, their aim was to reduce indiscriminate use of PEP following a shortage. Judicious use of PEP was considered a pivotal role for some IBCM programs (e.g., Bali, Haiti, Philippines), while being of minimal importance to others (e.g., Goa, India). In general, countries where frequent PEP shortages occur and/or governments pay for PEP placed more emphasis on its judicious use. Though, that was not always the case. Both India and the Philippines have high numbers of patients receiving government supplied PEP for healthy animal bites. Yet, in India, where they affordably manufacture human rabies biologics, there was no aim to reduce unnecessary PEP. Furthermore, certain country contexts made implementing IBCM challenging. For example, in India where unowned and fairly homogeneous-looking dogs limited the ability to trace animals. Program success was also hindered in extremely resource-poor countries (e.g., Chad, Madagascar) due to inadequate infrastructure and more pressing priorities. Alternatively, some contexts made implementing IBCM more straightforward, such as countries in Latin America (e.g., Brazil, Peru) with substantial budgets for mass dog vaccination, and a strong history of One Health in action facilitating successful rabies control.

This study underscores the complexity of IBCM which stems from the interaction of its many components as they relate to the context or system where they are implemented (55). These findings demonstrate the importance of transferring the evidence base of the IBCM approach to inform adaptations when implemented in a new context to ensure effectiveness (56). Tools such as the ADAPT guidance provide a framework and checklist, facilitating the streamlining of these processes and reducing research waste (57). To prevent misunderstanding of the concept of IBCM, future guidance should include an explicit program theory (58), articulating how IBCM is expected to contribute to a chain of intermediate results and ultimately to expected outcomes (via a Theory of Change, similar to Figure 2). This has the potential to illustrate to stakeholders how implementing IBCM can be useful in their own context and might help overcome challenges specific to their setting. Furthermore, guidance should be expanded to include clear examples of how IBCM has been operationalized in various settings and how activities, outcomes, and aims might differ accordingly. As well as, guidance should be provided for practitioners on the use of RDTs, and for health workers receiving patients to cover assessment of the history and signs of rabies in biting animals, which is not integrated into WHO guidance on post-exposure prophylaxis. Lastly, IBCM programs should use standardized WHO tools and practices (2)—tailored to local understandings—for interpreting rabies risk to improve data quality and comparability of the burden of rabies and transmission pathways between settings.

As a relatively new approach, IBCM exemplifies the challenges faced when implementing One Health (59), with lessons that could be applied to other complex zoonotic diseases. Integrated One Health approaches are vital for tackling both endemic and emerging zoonoses, and for antimicrobial resistance (60, 61). Strengthening surveillance systems in LMICs for endemic diseases, such as rabies, builds foundations to address emerging zoonotic diseases like COVID-19 (62). Formalizing One Health approaches through intersectoral government bodies, including joint budgets and policies, can help to overcome institutionalized/structural barriers (61, 62). Yet, like IBCM, varying perceptions about the concept of ‘One Health’ make it difficult to standardize the operationalization of these approaches in both high-income countries and LMICs (63, 64). These challenges are amplified by the lack of sustainability of funding and infrastructure that are exacerbated in LMICs. This study emphasizes the need for more implementation research to improve and understand IBCM program delivery and policies (65). In recent years, such studies have helped strengthen the gap between knowledge and real-world action for a variety of neglected tropical diseases (65, 66). Future research exploring the knowledge-practice gaps of implementing IBCM could improve the cost-effective roll out of IBCM and provide a potential example for other One Health interventions.

There are some limitations in this study. One-hour interviews provided limited time to discuss perspectives of and experiences with IBCM. For broader comprehension about the implementation and adaptation of IBCM, more in-depth qualitative research is required (e.g., ethnographic participant observations, development of program theory) (54). Caution is required regarding interpretation over the study’s reliability, as it was not designed to be representative for IBCM programs or specific regions/countries. Most participants had a background in veterinary medicine and/or research, and representation from the medical sector was lacking. Future research including the perspectives of clinicians and public health experts would be beneficial, however, it should be noted that there is relatively little involvement of medical professionals leading One Health programs for rabies. The logic model of IBCM (Figure 2) serves as a template, but does not fully describe the complexity of IBCM and how that relates to different contexts. Moreover, the programs included varied in maturity, from a decade in Bali, Indonesia (2011) to programs still in development, limiting direct comparison. Nevertheless, this study covered a wide scope of perspectives, work experience, epidemiological contexts (from elimination to high endemicity), and geographies. Hence, the study is a valuable step towards discovering lessons about this approach and for understanding how One Health can be operationalized to achieve dog-mediated rabies elimination.

We conclude with preliminary recommendations to support the design and implementation of IBCM programs keeping sustainability in mind. As the only endemic zoonosis with an official elimination goal set by the WHO, rabies should be prioritized and funded to support hiring of staff and implementation of control programs (15). A One Health approach to surveillance should be implemented in all endemic countries at any stage of their control program as this is the most targeted way to identify rabid animals. Many existing reporting systems for rabies are not fit-for-purpose for surveillance and provisioning of PEP. Surveillance systems are often siloed and do not consider the risk of the biting animal or recognize the value of risk assessments, leading to uninformed administration of PEP. In response, IBCM has been developed as a cost-effective approach to address these weaknesses (18). However, current structures within governments, policies, and ways of working pose barriers to introducing IBCM. To successfully and sustainably implement IBCM, there needs to be consideration for how governments and policy can be updated to better facilitate multisectoral, interdisciplinary approaches generally (67). It is imperative that each program is tailored to the context of the country/region where it will be implemented, with careful consideration of context during development, implementation, and evaluation (53). Lastly, the joint involvement and ownership of government authorities from all relevant sectors, local stakeholders, and the community is essential for the effective development and adoption of IBCM protocols (68, 69).

## Figures and Tables

**Table 1:** Economic and epidemiological context of countries included in this study.

Country	Continent	Population (2020)	GDP (2020, US $/Bn)	HDI (2019)	Rabies elimination stage	Level of dog vaccination	Owned, free roaming dogs	Deaths per year	Policy on PEP (cost per course)	Bite incidence (per 100k)	Estimated HDR ***	Source
Chad	Africa	16,425,860	10.1	0.40	Endemic	Not routine	>90%	550	Patient pays* (US$ 80100)	480-570	1: 7.8 (1: 5.2-40)	(10, 22, 23, 24)
							>90%					
Kenya	Africa	53,771,300	98.8	0.60	Endemic	Not routine	>90%	2,200	Patient pays* (US$ 85)	290	1: 4-8	(22, 25)
							>90%					
Madagascar	Africa	27,691,020	13.7	0.53	Endemic	Not routine	>90%	1,000	Free	190	1: 8-25	(26, 27)
							>90%					
Malawi	Africa	19,129,955	12.0	0.48	Endemic	Not routine	>90%	900	Free	230	1: 23 (1: 14-31.8)	(22, 28)
							>90%					
Tanzania	Africa	59,734,210	62.4	0.53	Endemic	Not routine	>90%	650	Patient pays (>US$ 80)	12-120	1: 20.7 (1: 7-181.3)	(9, 22, 29)
							>90%					
Brazil	Americas	212,559,410	1,445.0	0.77	Elimination*	Routine*	>90%	<10	Free	230-280	1: 4.2-7	(30, 31)
							<50%					
Guatemala	Americas	16,858,330	77.6	0.66	Endemic	Routine	>80%	0-8	Free	150-280	1: 6.4 (1: 1-10)	(30, 33, 34)
							>70%					
Haiti	Americas	11,402,530	13.4	0.51	Endemic	Routine	>90%	350	Free	200	1: 5.2	(22, 35)
							>50%					
Peru	Americas	32,971,850	202.0	0.78	Emerging*	Routine	>80%	0-10	Free	200-600	1: 3.8	(30, 36, 37)
							>40%					
India	Asia	1,380,004,390	2,623.0	0.65	Endemic *Goa State - elimination	Highly variable	>60%	>15,000 *Goa State: 0 since 2018	Free	1,300	1:11-36	(22, 38, 39)
							>40%					
Indonesia	Asia	273,523,620	1,058.0	0.72	Endemic*	Not routine	>90%	3,300	Free	200	1: 8.3-360	(22, 41, 42)
							>70%					
Philippines	Asia	109,581,090	361.5	0.72	Endemic	Routine but variable	>80%	200-300	Free	1,100	1:4-10	(43, 44, 45)
							>50%					
Vietnam	Asia	97,338,580	271.2	0.70	Endemic	Routine but variable	>80%	500	Patient pays (US$ 150)	400	1: 10-38	(22, 46, 47, 48)
							>50%					

GDP, Gross Domestic Product, HDI, Human Development Index, PEP, post-exposure prophylaxis, HDR, Human to Dog Ratio. Population and GDP data from the World Bank OECD National Accounts data files, 2020 (https://data.worldbank.org). HDI data from the United Nations Development Programme 2020 Human Development Index Ranking (https://hdr.undp.org). Rabies elimination stage, level of rabies control, and policy on PEP were all reported from interviews. Deaths per year, annual bite patient incidence, and estimated human:dog ratios were from the literature.

* Brazil is close to elimination, but rabies has continuously circulated in the state of Maranhao (47).

* Peru is close to elimination, but rabies has continuously circulated in the border state of Puno and re-emerged in the city of Arequipa in 2015 (36). Canine rabies is endemic in India, but the State of Goa is now close to elimination and has not had a human rabies death since 2018 (47). Indonesia has endemic dog rabies in 26 provinces, while 8 provinces are rabies-free (39).

* Dog vaccination is routine in 24/27 Brazilian states, but has been discontinued in 3 southern states: Parana', Santa Catarina and Rio Grande do Sul. There is considerable variability in the degree of routine dog vaccination reported in Asian countries.

* Patients pay for PEP in Chad, but PEP was provided for free during the IBCM project (2016-2018).

* Patients pay for PEP in most of Kenya, but PEP is free in a few counties (e.g., Makueni). Bite patient incidence (presentations to health facilities) is reported rather than cross-sectional surveys which typically are much higher.

*** Variability in HDRs relates to culture (with major differences between religions).

**Table 2:** Comparison of operationalized IBCM programs.

Country	Region of IBCM program	IBCM start and end dates	IBCM funding source	Mobile application used	Hired team vs local govt staff	Where bite events are reported	Who does risk assessment	How investigation is triggered	Who does animal investigation	Rapid Diagnostic Tests used
Chad	4 regions*	2016-2018	Research	✘ No app	Local govt + hired hotline staff	Hotline	Govt health workers + hired hotline staff	Hotline staff call investigator	Govt animal health staff	✔ Yes
Kenya	Makueni county	To start 2021/ 2022	Research	✔ App	Local govt + hired field/ hotline staff	Health facilities + hotline	Govt health workers + hired field / hotline staff	App notification &/ call/SMS to investigator or same person	Govt animal health staff + hired field staff	✔ Yes
Madagascar	Moramanga District	2016-2019	Research	✔ App	Local govt + 1 hired field staff	Health facilities	1 hired field staff at 1 clinic*	Call/SMS to investigator	Govt animal health staff + Govt health worker	✘ No
Malawi	Districts of Blantyre, Zomba and Chiradzulu	2018 - present	Research, Donor	✔ App	Local govt + hired field/ hotline staff	Hotline + health facilities	Govt health workers + hired field/ hotline staff	Call/SMS to investigator	Govt animal health staff + hired field staff	✔ Yes
Tanzania	20 districts in 4 regions*	2018 - present	Research	✔ App	Local govt + 2 hired field staff	Health facilities	Govt health workers	Call/SMS or group chat message to investigator	Govt animal health staff	✔ Yes
Brazil	State of Rio Grande Do Sul*	2015 - present	Govt	✘ No app	Local govt	Health facilities	Govt health workers	Call investigator	Govt animal health staff	✘ No
Guatemala* *TBC*	TBD	Pilots started in 2020	Donor, Govt	✔ App	Local govt + hired field staff	Health facilities + community outreach*	Govt health workers	App notification &/call/SMS to investigator	Hired field staff or local vet students*	✘ No
Haiti	7 of 10 departments	2013 - present	Donor, Govt	✔ App	˜15 hired field staff	Health facilities + community outreach*	Hired field staff	Same person assesses risk then investigates	Hired field staff	✘ No
Peru* TBC	Regions of Arequipa and Puno	Pilots started in 2019	Donor, Govt	✔ App	Local govt + >20 hired field staff	Health facilities + community outreach*	Govt health workers	App notification &/call/SMS to investigator	Hired field staff	✘ No
India	State of Goa	2018 - present	Donor, Govt	✔ App	˜20 hired hotline & field staff	Hotline + health facilities	Hired hotline staff	Hotline staff notify field staff	Hired field staff	✔ Yes
Indonesia	Province of Bali	2011 - present	Donor, Govt	✘ No app	Local govt	Health facilities	Govt health workers	Call/SMS to investigator	Govt animal health staff	✘ No
Philippines	Province of Albay	2017-2021	Research	✔ App	Local govt + 3 hired field staff	Health facilities	1 hired field staff per clinic	Call/SMS to investigator	Govt animal health staff	✘ No
Philippines	Provinces of Romblon & Oriental Mindoro	2019 - present	Govt, Research	✔ App	Local govt	Health facilities	Govt health workers	Call/SMS or group chat message to investigator	Govt animal health staff	✔ Yes
Vietnam	5 provinces*	2019 - present	Donor, Govt	✔ App	Local govt	Health facilities	Govt health workers	App notification &/group chat message to investigator	Govt animal health staff	✘ No

* IBCM programs in Guatemala and Peru have started training and pilot studies, but implementation was delayed due to COVID-19. IBCM is not being used yet and both countries are still relying on passive surveillance to find rabies cases.

* Chad’s IBCM program was implemented in 4 administrative regions: Logone Occidentale, Ouaddaï, Hadjer Lamis, and Chari Baguirmi (21). Tanzania’s IBCM program is implemented in 4 regions: Mtwara, Mara, Lindi, and Morogoro (13). Brazil does not have an official IBCM program, but similar protocols are implemented in 3 states in the South Region: Paraná, Santa Catarina and Rio Grande do Sul. Vietnam’s IBCM program is currently implemented in 5 provinces in Central and Northern Vietnam: Phú Thọ, Bà Rịa-Vũng Tàu, Nghệ An, Lạng Sơn and Đắk Lắk and will be expanded to a further 4 provinces in 2022, which includes Southern Vietnam. Information collected through interview data.

**Table 3:** Barriers and facilitators to implementation of IBCM programs.

	Risk Assessment	PEP Provisioning	Animal Investigation	One Health collaboration	Data reporting/mobile technology
**Barriers**	**Govt health workers:** Work Capacity Busy workload High patient volume Many responsibilities Other priorities Work duplication Human & Financial Resources High staff turnover Not compensated for additional IBCM work Compliance with guidelines Felt rabies not their job Reluctance to change or adopt new routine Performing risk assessment Limited knowledge of rabies incl. WHO Categories & signs of rabies in animal Focus on wound severity not rabies signs in biting animal Judicious use of PEP Hesitancy to discontinue or withhold PEP No legal basis for risk assessment to inform PEP	**PEP accessibility:** Costs High out-of-pocket costs of PEP High travel costs to closest clinic w/ PEP Access Most vulnerable populations don't have access to health services **PEP availability:** Frequent stockouts Not available in many rural/ remote areas **Excessive PEP use:** Free PEP policy can lead to excessive PEP-seeking behavior Indiscriminate PEP use can cause excessive costs/ stockouts Same or more demand for PEP even when low or zero risk of rabies **Local beliefs:** Seeking care at traditional healers	**Govt animal health workers:** Compliance with guidelines Investigations late or not conducted No follow-up Reluctance to change or adopt new routine Felt rabies not their job Other prioritizes Not motivated to investigate until death Human & Financial Resources Lack of personnel High staff turnover Not compensated for time or travel costs Training | Lack of formal trainingl Not comfortable handling animals **Conducting animal investigation:** Cannot find or identify biting animall Not able to get sample **Diagnostics:** Few diagnostic lab(s)| Must ship samples far without cold chain	**National-level:** Governance structures| Govt ministry not structured for One Health| Lack of ministry cooperation| Unbalanced sector power Policy | Rabies neglected/not priority Lack of funding Difficult integrating IBCM into national policy **Regional / Local-level:** Compliance with guidelines Health sector not notifying about bites Investigation results not considered for PEP decision Stakeholder Engagement Lack of local ownership No prior intersectoral communication Difficult to change routine behavior Difficult to establish and maintain communication Required Skills Limited experience with data management	**Data reporting:** Lack of data submission by both sectors Feedback not provided to other sectors Investigation case not formally closed **Mobile technology:** App issues App development timely and many iterations App not feasible in all settings – e.g., high illiteracy/no written language Limited network coverage App must be updated and re-downloaded User Issues Lack access to smartphones/tablets Reluctance to use app Required additional proficiency training
**Facilitators**	**Workforce:** Hiring staff to perform risk assessments Consistent feedback and communication **Training & Materials:** Apps that automatically assign case definition >Provision of hard copy protocols Routine training	**Free PEP policy:** Increased accessibility Increased PEP-seeking Increased PEP adherence **Community Outreach:** Educating local traditional healers to report bites	**Workforce:** Hiring staff to conduct animal investigations **Training & Materials:** Hook and straw sampling techniques Rapid diagnostic tests Safely handling animals **Communication:** Prepare stakeholders for swift rise in case detection	**Communication:** Consistent feedback with all involved sectors Establishing hotline to facilitate One Health link **Collaboration:** Involving all sectors in discussions/decisions Using existing One Health programs and networks Employing veterinary staff within public health system	**Communication:** Consistent feedback/ reminders to report **Mobile technology:** Real-time data monitoring Remote data access Quick way to establish a surveillance system Overcome language barriers

## Data Availability

Data supporting the conclusions of this article will be made available on request due to privacy/ethical restrictions.

## Abbreviations

FAO: Food and Agriculture Organization
GARC: Global Alliance for Rabies Control
IBCM: Integrated Bite Case Management
LMIC: low- and middle-income countries
OIE: World Organisation for Animal Health
PEP: post-exposure prophylaxis
RDT: rapid diagnostic test
WHO: World Health Organization.
